# A Mediated Enzymatic Electrochemical Sensor Using Paper-Based Laser-Induced Graphene

**DOI:** 10.3390/bios12110995

**Published:** 2022-11-09

**Authors:** Panpan Gao, Toshihiro Kasama, Jungchan Shin, Yixuan Huang, Ryo Miyake

**Affiliations:** 1 Microfluidic Integrated Circuits Research Laboratory, Bioengineering, School of Engineering, The University of Tokyo, Tokyo 113-8656, Japan; 2 Bioengineering, School of Engineering, The University of Tokyo, Tokyo 113-8656, Japan

**Keywords:** paper-based sensor, laser-induced graphene, two-step laser engraving, second generation glucose biosensor

## Abstract

Laser-induced graphene (LIG) has been applied in many different sensing devices, from mechanical sensors to biochemical sensors. In particular, LIG fabricated on paper (PaperLIG) shows great promise for preparing cheap, flexible, and disposable biosensors. Distinct from the fabrication of LIG on polyimide, a two-step process is used for the fabrication of PaperLIG. In this study, firstly, a highly conductive PaperLIG is fabricated. Further characterization of PaperLIG confirmed that it was suitable for developing biosensors. Subsequently, the PaperLIG was used to construct a biosensor by immobilizing glucose oxidase, aminoferrocene, and Nafion on the surface. The developed glucose biosensor could be operated at a low applied potential (−90 mV) for amperometric measurements. The as-prepared biosensor demonstrated a limit of detection of (50–75 µM) and a linear range from 100 µM to 3 mM. The influence of the concentration of the Nafion casting solution on the performance of the developed biosensor was also investigated. Potential interfering species in saliva did not have a noticeable effect on the detection of glucose. Based on the experimental results, the simple-to-prepare PaperLIG-based saliva glucose biosensor shows great promise for application in future diabetes management.

## 1. Introduction

As one of the greatest threats to global health, it is predicted that around 642 million people will be living with diabetes by 2040 [1]. Diabetes is a chronic disease in which the patient has a high blood glucose concentration outside the normal range [1]. For the management of diabetes, diabetic patients are required to perform blood glucose testing every day to enable more accurate titration of their insulin dose [2,3]. In recent years, in place of invasive blood glucose monitoring, more and more researchers are working on the development of non-invasive approaches to improve the lifestyle of diabetic patients that can preclude the need for frequent skin punctures [4,5,6]. Based on previous studies, many biofluid samples can also be used to indicate the glucose levels in the body, and saliva is a good candidate [4,5,6]. Saliva is an easily accessed biofluid sample produced by the parotid gland, and it has been reported that the saliva glucose level shows a strong correlation with the blood glucose level [6,7,8]. Hence, the development of easily disposable or burnable saliva glucose sensors holds great promise for the future of diabetes management. It was reported that the saliva glucose concentration ranges from 0.23–0.38 mM for healthy people, while for diabetic patients it is 0.55–1.77 mM [5]. Compared with the glucose level in the blood (4.9–40 mM) [5], such a low glucose concentration in saliva requires good sensitivity to produce a viable saliva glucose biosensor. For glucose measurement, there are mainly two types of glucose biosensors: enzymatic and non-enzymatic [1]. For glucose measurement, enzyme-based electrochemical measurement is one of the most widely investigated methods, because of their great accuracy, low cost, and simple design [1,4,9,10]. Nevertheless, the performance of an enzymatic biosensor can be affected by temperature, pH, humidity, and other toxic elements [1]. As a result, numerous efforts have been made to develop a non-enzymatic glucose biosensor based on nanomaterials, such as the Cu and Pt nanomaterials [1]. However, non-enzymatic glucose biosensors are suffered from poor selectivity due to the absence of a selective recognition element in their structure [1]. In addition, the majority of non-enzymatic glucose biosensors rely on an alkaline environment, which restricts their practical application [1]. Therefore, from the perspective of practical use and commercialization, enzymatic-based biosensors are still the majority of glucose biosensors. For the fabrication of enzyme-based electrochemical biosensors, the properties of the electrode material, such as the conductivity and surface area, strongly affect the performance of the biosensor.

Several types of wearable saliva glucose biosensors have been developed in recent years, including the mouthguard type and the pacifier type [11,12]. Nevertheless, the sensing electrode parts of the many developed saliva biosensors are based on screen-printing or metal deposition on a plastic sheet, which may not satisfy the need for an easily disposable sensor. Since it was first fabricated in 2014, laser-induced graphene (LIG) on polyimide has been investigated by many researchers because of its easy fabrication and good physical and chemical properties [13,14]. The LIG material generated on polyimide film (PILIG) shows good conductivity and a high surface area, and has been applied to different kinds of biochemical sensors, such as uric acid sensors, dopamine sensors, and glucose sensors [14,15,16,17,18,19,20,21]. Nevertheless, for the PILIG-based biosensor, the disposal of the biosensor may burden the environment and cause pollution because polyimide is a type of synthetic polymer with great stability [22,23]. Compared with polyimide, paper made from cellulose fiber is a recyclable material with a lower cost. More importantly, paper can be easily disposed by simply gasifying it with low thermal energy. Previously, paper material has been reported to be suitable as a substrate for fabricating LIG, and this kind of LIG can be termed PaperLIG [24,25,26]. As an electrode material, PaperLIG can be fabricated in a simple and low-cost way through laser engraving the paper material using the commercialized CO_2_ laser system, which shows great potential for the development of low-cost and easily disposable biosensors. 

Previously, most of the reported LIG-based enzymatic glucose biosensors depended on oxygen as the electron acceptor, and the glucose level is indicated by measuring the H_2_O_2_ (hydrogen peroxide) produced by the reaction of enzymes, glucose, and oxygen [19,20,21,27]. This kind of glucose biosensor is also known as a first generation glucose biosensor [1,4]. The detection of H_2_O_2_ requires a high applied potential, which may introduce a noise signal coming from other redox-active species in the sample, such as ascorbic acid or uric acid [1,4]. To overcome this challenge, a synthetic mediator is applied in second generation glucose biosensors as an electron acceptor instead of oxygen [1,4]. The glucose level can be detected by measuring the concentration change of the synthetic mediator under the applied potential [1,4]. As an example, ferrocene and its derivatives are commonly used mediators in glucose biosensors due to their fast reaction with glucose oxidase, low overpotential for redox reactions, and reversible electron transfer kinetics [1,28,29]. 

**Figure 1:**
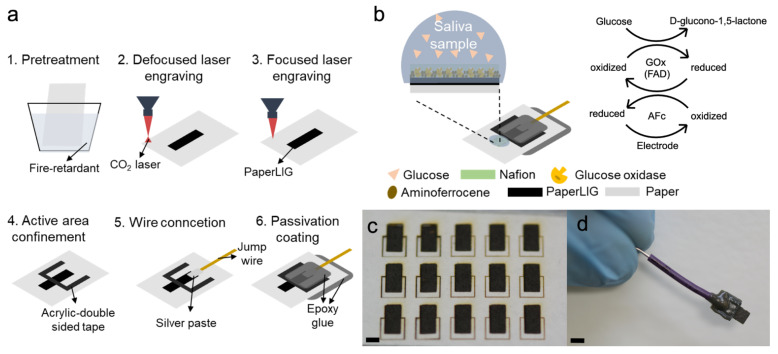
(**a**) Illustration of the fabrication process of PaperLIG electrode. (**b**) The structure of the developed PaperLIG-based glucose biosensor. The sensing mechanism of the glucose biosensor is also shown on the right. (**c**) Mass production of PaperLIG material. Scale: 3 mm. (**d**) The fabricated PaperLIG electrode. Scale: 3 mm.

In this study, we proposed a simple approach to construct an easily disposable glucose biosensor based on the PaperLIG material. As previously stated, many glucose biosensors based on non-disposable PILIG materials are well reported, but glucose biosensors based on the low-cost and disposable PaperLIG materials are rarely reported. More importantly, to our knowledge, this is the first reported second generation enzymatic glucose biosensor based on PaperLIG and an artificial mediator. In this research, we first fabricated a highly conductive PaperLIG material on filter paper. Characterization of the PaperLIG confirmed that it possesses a porous structure, which provides a high real surface area for the adhesion of ferrocene derivatives and enzymes. The deposited ferrocene derivatives with low solubility in water can function as the mediator layer for an enzymatic biosensor. The PaperLIG electrode was fabricated (Figure 1a) and further electrochemical characterization of the electrode proved its suitability for use as an electrochemical biosensor. Finally, PaperLIG was applied in the development of a glucose biosensor by drop-casting aminoferrocene (AFc), glucose oxidase (GOx), and Nafion on its surface (Figure 1b). As a perfluorinated sulfonated cation exchanger, Nafion coating can improve the stability of enzymatic biosensors and prevent possible interference from the other redox species in the saliva sample such as uric acid or ascorbic acid [29]. The influence of the concentration of the Nafion solution used to cast the film on the performance of the developed glucose biosensor was also investigated. The performance of the glucose biosensor was evaluated in the PBS and artificial saliva and the results indicate the great potential of PaperLIG-based glucose biosensors for diabetes management in the future.

## 2. Materials and Methods

### 2.1. Material and Chemicals

Filter paper (Whatman filter paper, Qualitative filter paper No.2, Cytiva, Marlborough, MA, USA) was purchased for the fabrication of PaperLIG. Fire retardants mainly composed of ammonium polyphosphate ([NH_4_PO_3_]_n_(OH)_2_) and ammonium sulfate ((NH_4_)_2_SO_4_) were purchased from Grow Chemical (Sabae, Fukui, Japan). Glucose oxidase, aminoferrocene (C_10_H_11_FeN), 1X PBS, potassium chloride, and glucose were all purchased from Wako Chemicals (Fujifilm Wako Chemicals, Osaka, Japan). Potassium ferricyanide (iii) (K_3_Fe(CN)_6_), potassium ferricyanide (ii) (K_4_Fe(CN)_6_), and Nafion (5% in a mixture of lower aliphatic alcohols and water, (contains 45% water) were purchased from Sigma Aldrich (Burlington, MA, USA). Artificial saliva [30,31] (SAE0419, Artificial Saliva for Pharmaceutical Research, pH: 6.8, main composition: NaCl, KCl, KSCN, KH_2_PO_4_, urea, and distilled water) was purchased from Sigma Aldrich. Conductive silver paste (CR-2800) was purchased from Kaken (Kaken Tech, Katano, Osaka, Japan). Epoxy glue (Araldite 2014) was purchased from Huntsman (Huntsman Corporation, The Woodlands, TX, USA). An acrylic double-sided tape (5620BWN, Nitto Nitoms, Tokyo, Japan) was used to confine the active area of the PaperLIG electrode. All reagents were used without further purification. Millipore water (Direct-Q^®^ Ultrapure water type 1, Millipore, Burlington, MA, USA) was used for preparing the solutions used in the experiment unless otherwise specified. Ethanol was used to dissolve the aminoferrocene.

### 2.2. Fabrication of PaperLIG Electrode

A simple schematic of the fabrication process of the PaperLIG electrode is shown in Figure 1. The patterns of the PaperLIG, PaperLIG electrode, and double-sided tape were all designed using CorelDRAW software (Corel Corporation, Ottawa, ON, Canada). A 10.6 µm CO_2_ laser system (VLS 3.5 Laser system, Universal Laser Systems, Scottsdale, AZ, USA) was used to engrave the filter paper to fabricate the PaperLIG material. The filter paper was firstly cut and put into the fire retardant solution for 2 min and then it was dried in the oven at 60 °C for 10 min. After drying, the filter paper was attached to a glass substrate and started the laser engraving. A two-step laser engraving process was applied for the PaperLIG fabrication. The filter paper was firstly engraved using the laser system with 2.5 W power, 3.5 inch/s speed, 1000 PPI, HD lens (spot size: 0.03 mm), and +5 mm defocus from the laser focal plane. Then, the filter paper was engraved with 2.5 W power, 4–9.5 inch/s speed, 1000 PPI, and HD lens at the focal plane. Then, the fabricated PaperLIG (3 × 6 mm^2^) was firstly rinsed with the DI water and then it was dried in the vacuum condition. In order to confine the electrode active area (3 × 3 mm^2^), the acrylic double-sided tape was cut by the laser system with 8.5 W power, 5 inch/s speed, 1000 PPI, and HD lens at the focal plane. The patterned acrylic double-sided tape was then attached to the surface of the PaperLIG electrode and, after confinement, the active surface area of the PaperLIG electrode was 3 × 3 mm^2^. Wire contacts were then applied to the confined PaperLIG electrode using the silver paste, and the paste was cured in a 90 °C drying oven for 60 min. After that, epoxy glue was coated on the silver paste and the backside of the filter paper to serve as an insulating barrier to avoid direct contact between the samples and the silver paste.

### 2.3. Characterization of the PaperLIG Material

Raman spectroscopy (XploRA PLUS, HORIBA, Kyoto, Japan) was used to determine the structure of the obtained carbon-based material. EDS (Quanta 250/EDS, FEI, Hillsboro, OR, USA) and XPS (X-ray Photoelectron Spectroscopy) (JPS-9010MS, JEOL, Tokyo, Japan) were used to analyze the elemental composition of the PaperLIG and filter paper. Scanning electron microscopy (VE-8800, Keyence Corporation, Osaka, Japan) was used to observe the surface structure of the PaperLIG material. For the sheet resistance measurement, square-type PaperLIG materials (5 × 5 mm^2^) were manufactured, and then the sheet resistance was measured by the four-point probe method using a digital multimeter (Agilent 34410A, 6 1/2 digital multimeter, Agilent Technologies, Santa Clara, CA, USA) and a four-point probe (BAS, Tokyo, Japan). For the measurement, the linear array four-point probe made soft contact with the center part of the fabricated PaperLIG material.

### 2.4. Electrochemical Measurement of the PaperLIG Electrode

Electrochemical characterization of the fabricated PaperLIG electrode (area: 3 × 3 mm^2^) was carried out using a Gamry 600+ potentiostat (Gamry, Philadelphia, PA, USA). The platinum counter electrode and Ag/AgCl reference electrode for the electrochemical measurements were obtained from BAS. Cyclic voltammetry (CV) and electrochemical impedance spectroscopy (EIS) were used to characterize the fabricated PaperLIG electrode. 

### 2.5. Preparation and Evaluation of Enzymatic Glucose Biosensor

A 0.1 M AFc solution was prepared by dissolving AFc powder in ethanol. A 20 mg/mL GOx solution was prepared by dissolving the GOx power in a 1X PBS solution. The Nafion solution (0.25%, 0.5%, 0.75%, and 1.0%) was prepared by diluting the 5% Nafion using a 1X PBS solution. Modification of the PaperLIG electrode was carried out as shown below. Previously, researchers employed a similar drop-casting method for the ferrocene-based glucose biosensor on a glassy carbon electrode (geometric area: 0.12 cm^2^) [28]. Considering the poorer conductivity and highly porous surface of PaperLIG in comparison to a glassy carbon electrode, a higher amount of AFc was used for preparing the PaperLIG-based glucose biosensor. An amount of 5 µL of the 0.1 M AFc solution was firstly drop-casted on the PaperLIG electrode, and then the PaperLIG electrode was dried under ambient conditions for 15 min. Then, 5 µL of the 20 mg/mL GOx solution was drop-casted on the PaperLIG electrode and dried in a 4 °C refrigerator overnight. Then, 4 µL of 0.25–1.0% Nafion solution was drop-casted on the dried PaperLIG electrode, followed by drying under ambient conditions for 1 h. The as-prepared PaperLIG-based glucose biosensors were stabilized in the PBS for at least 2 h before use. The performance of the developed PaperLIG-based glucose biosensors was evaluated in the PBS using the amperometry method. The glucose concentration was adjusted by adding 0.1 M glucose solution into the PBS by pipette with magnetic stirring.

## 3. Results

### 3.1. Fabrication and Characterization of PaperLIG Material

**Figure 2:**
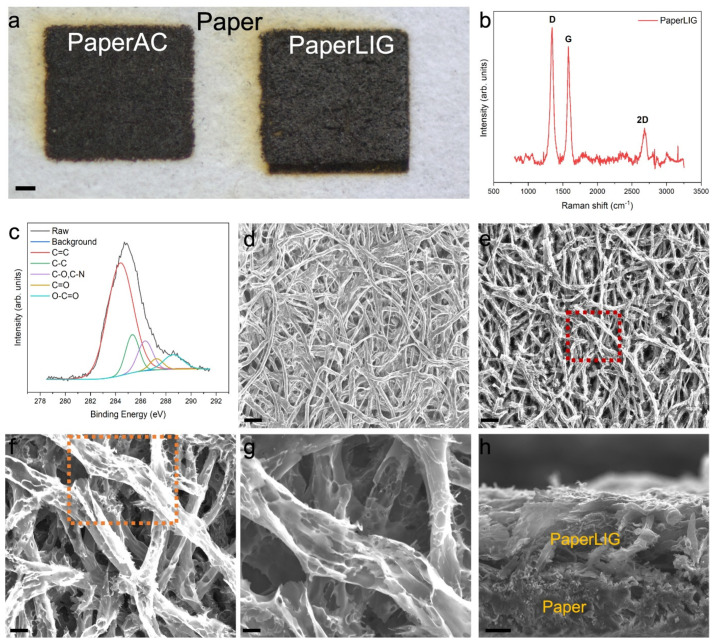
(**a**). The optical image of fabricated PaperAC and PaperLIG. Scale: 1 mm. (**b**) Raman spec-trum of PaperLIG material. Three prominent peaks are found in the Raman spectrums. (**c**) The high-resolution C1s XPS spectrum of PaperLIG. The raw curve was fitted with peaks corresponding to five carbon species. (**d**,**e**) SEM images of filter paper and PaperLIG. Scale: 100 µm. (**f**). A magnified SEM image of the red rectangular part of (**e**). Scale: 20 µm. (**g**) A magnified SEM image of the orange rectangular part of (**f**). Scale: 10 µm. (**h**) A cross-sectional SEM image of PaperLIG. Scale: 33.3 µm.

In order to fabricate easily disposable PaperLIG material, the filter paper was chosen as the substrate for laser processing. Unlike many office paper or food packing paper, which contains nonvolatile components or polymer coating [32,33], filter paper is composed of 100% cellulose fiber, making it an ideal substrate for the fabrication of disposable PaperLIG material. However, unlike many synthetic polymers, cellulose fiber cannot withstand high temperatures and is easily volatilized during laser processing [24,25,26]. To avoid possible volatilization, pretreatment of the paper with a fire retardant is necessary [24,25,26]. A fire retardant is a type of chemical that can prevent the volatilization of easily burnable material [34]. The fire retardant used in this study was an environmentally friendly type with no boron content. Similar to PEEK and PEI polymers, paper can be transformed to a LIG material by a multiple laser-engraving process, which is realized by a repeated laser engraving method or defocused laser engraving method [24]. By utilizing the multiple laser-engraving method, mass production of LIG material on paper can be accomplished simply (Figure 1c). It was reported that during the multiple laser-engraving process, the paper was first transformed into amorphous carbon (AC) and then further laser engraving could transform the AC to LIG [24,25]. As Figure 1 shows, in this study, a two-step process was applied for the fabrication of PaperLIG. After pretreatment of the paper with a fire retardant, the paper is first transformed to paper-based amorphous carbon (PaperAC) by defocused laser engraving (5 mm below the focal plane), and then the obtained PaperAC (Figure 2a at left) is transformed to PaperLIG (Figure 2a at right) by focused laser engraving. The colors of the fabricated PaperAC and PaperLIG are distinct from the white color of the pristine paper. PaperLIG shows a slightly gray appearance, while PaperAC shows a darker color (Figure 2a). 

The Raman spectrum of the material obtained after the two-step laser engraving is shown in Figure 2b. Three predominant peaks are found in the Raman spectrum: the D peak at ~1344 cm^−1^, the G peak at ~1580 cm^−1^, and the 2D peak at ~2690 cm^−1^, which are all similar to the results for LIG materials and other graphene-based materials in previous reports [19,20,35] and suggest the formation of graphene material. As a comparison, the Raman spectrum of the PaperAC material after the first defocused laser engraving is shown in Figure S1 and it presents the characteristic spectrum of amorphous carbon [25]. An EDS analysis can be used to determine the compositional information of the prepared PaperLIG material. As shown in Figure S2, the main components of PaperLIG are carbon, nitrogen, oxygen, phosphorus, and sulfur, which are different from the composition of cellulose-based paper material; the nitrogen, phosphorus, and sulfur are contributed by the fire retardant. After the laser engraving, the carbon content increases significantly compared with the original paper material (Figure S2 and Table S1). The release of the recombined oxygen atoms after two-step laser engraving of the paper material can be confirmed by the change in the elemental composition of PaperLIG compared to paper (Table S1). The XPS spectrum can be used to indicate the chemical state of atoms on the surface of the obtained PaperLIG material. The high-resolution C1s XPS spectrum of PaperLIG (Figure 2c) indicates that there are five main forms of carbon species: C=C, C-C, C-O/C-N, C=O, and O-C=O. The peaks of different carbon species in the high-resolution C1s spectrum were fitted and adjusted according to the analysis given in the previous study [24,25,26]. 

The surface structure of PaperLIG was investigated by SEM observation. As shown in Figure 2d, before laser engraving, a long cellulose fiber structure with a well-connected fibrous network structure can be observed from the SEM image. After the transformation to PaperLIG, the long and connected fiber structure is found to be broken into shorter pieces (Figure 2e), which may be caused by the highly localized temperature of the laser engraving [24,25]. In addition, the surface of PaperAC still presents a structure consisting of long fibers and a fibrous network (Figure S3a). A broken fiber structure with a slightly porous surface on each fiber can be observed from the magnified SEM image of PaperAC (Figure S3b). A magnified SEM of the red rectangle part of Figure 2e is shown in Figure 2f and a highly porous surface can be observed for each fiber (Figure 2f). The highly porous and graphene flake-like structure can be observed clearly from a further magnified SEM (Figure 2g), which is similar to the SEM image of LIG derived from polyimide [13]. The porous structures of each fiber of PaperAC and PaperLIG are generated by the liberation of the recombined oxygen content as a gas, which matches with the EDS analysis (Table S1) [24]. Such a porous graphene structure on each fiber of PaperLIG indicates that it has a large real surface area, which can provide a large area for the immobilization of enzymes and mediators. The cross-sectional SEM of PaperLIG (Figure 2h) also demonstrates the porous and graphene flake structure on its surface, with the thickness of PaperLIG being 89 ± 2 µm. The long fiber structure of PaperAC is also confirmed by the cross-sectional SEM image (Figure S3d).

### 3.2. Characterization of PaperLIG Electrode

**Figure 3:**
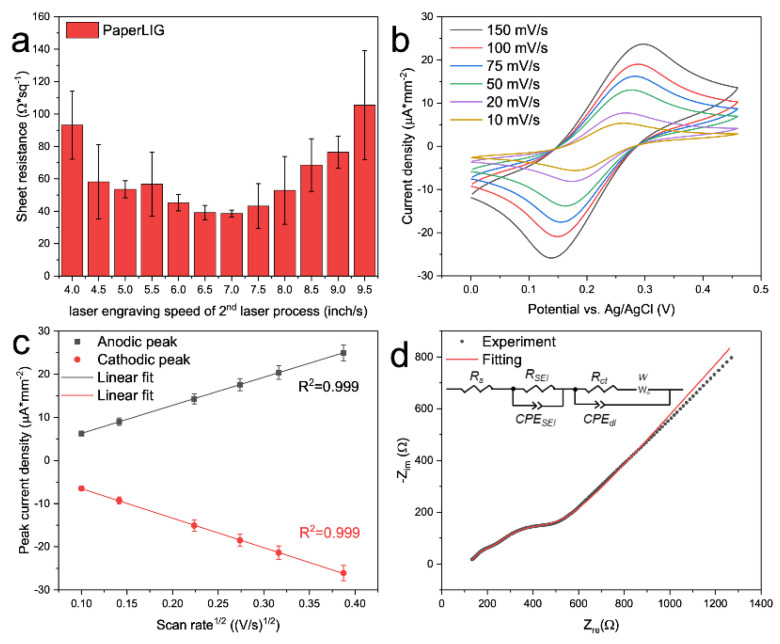
(**a**) Sheet resistance measurement of PaperLIG (*n* = 10) fabricated with various laser en-graving speeds of second laser processing. (**b**) Cyclic voltammetry of PaperLIG electrode in the PBS solution containing 5 mM K_3_Fe(CN)_6_/K_4_Fe(CN)_6_ with 0.1 M KCl under different scan rates (10–150 mV/s). (**c**) The plot of peak current density (*n* = 10) versus the square root of the scan rate for the CV of PaperLIG electrodes (**b**). (**d**) Nyquist plot of EIS of PaperLIG electrode in the PBS solution containing 5 mM K_3_Fe(CN)_6_/K_4_Fe(CN)_6_ with 0.1 M KCl. The inset shows the equivalent circuit used for fitting the experiment result.

After the successful fabrication and characterization of the PaperLIG material, the PaperLIG was applied as an electrode material (Figure 1d). As an electrode material, the conductivity of the PaperLIG is an important parameter, and the conductivity of PaperLIG can be evaluated by measurement of the sheet resistance [13,24,25,26]. During the laser engraving process, the laser setting can affect the photothermal effect of the engraving process, which can affect the quality of the obtained PaperLIG material. Especially for PaperLIG, a two-step process is required for the fabrication. After the first defocused laser engraving, the paper is transformed into PaperAC. The second step involves a focused laser engraving process that can transform the PaperAC into PaperLIG [24,25]. In this study, we investigated the effect of the speed of the second laser engraving step on the quality of the PaperLIG. For this purpose, after the first laser processing step, the obtained PaperAC was laser engraved with the second laser at different speed settings (4–9.5 inch/s) at a fixed laser power of 2.5 W, PPI of 1000, and in the focal plane. As shown in Figure 3a, as the laser engraving speed is increased, the sheet resistance value of the obtained PaperLIG decreases to a lower value before increasing again, which corresponds to the diverse photothermal effects generated by different speed settings. The photothermal effect caused by laser fluence can be simply explained by Equation (S1) [36,37]. Based on our experiment, the optimized second laser engraving speed is 7 inch/s with a minimum sheet resistance of 38.64 ± 2.11 Ω·sq^−1^, which is comparable to the lowest sheet resistance reported for PaperLIG material [25,26]. Decreasing the engraving speed from the optimal setting may lead to over-ablation of the graphene structure and generate an overabundance of photothermal energy [13]. Under ambient conditions, over-ablation of LIG can cause the re-oxidation of the produced LIG material, which degrades the quality of graphene material and thus increases the measured sheet resistance [13]. Meanwhile, further increasing the engraving speed resulted in a weak photothermal effect, which was not sufficient to fully convert the PaperAC to PaperLIG, and thus a higher sheet resistance was obtained [13]. 

After determining the optimized setting for the fabrication of PaperLIG, the highly conductive PaperLIG electrode was fabricated. Before the development of the glucose biosensor, the performance of the PaperLIG electrode was evaluated by electrochemical measurements. A typical three-electrode setup was used for electrochemical characterization. The PaperLIG electrode was used as the working electrode, a platinum electrode as the counter electrode, and a glass Ag/AgCl electrode as the reference electrode. Cyclic voltammetry at various scan rates (10–150 mV/s) was carried out in the PBS solution containing 5 mM K_3_Fe(CN)_6_/K_4_Fe(CN)_6_ with 0.1 M KCl. As Figure 3b shows, the peak separation between the anodic and cathodic peaks decreased with decreasing scan rate, which indicates a quasi-reversible redox reaction [38,39]. Plots of the peak current density (Figure 3b) versus the square root of the scan rate are shown in Figure 3c (*n* = 10). According to the Randles–Sevcik equation (Equation (S2)), for the electrochemically reversible electrochemical process involving freely diffusing redox species, the peak current of CV should increase linearly with the square root of the scan rate [38]. As a reference, the plots of the peak current density versus scan rate were also obtained (Figure S4a) and the poorer linearity of the plots could be observed. The good linearity of the plot in Figure 3c indicates that the mass-transfer process of [Fe(CN)6]^3−/4−^ on the PaperLIG electrode is mainly diffusion-controlled [38]. Based on the Randles–Sevcik equation (Equation (S2)), the electroactive surface area (ECSA) could be estimated from Figure 3c [38,40,41,42]. For the used K_3_Fe(CN)_6_/K_4_Fe(CN)_6_, *D* (diffusion coefficient) was equal to 6.67 × 10^−6^ cm^2^s^−1^ (for anodic peak), *n* was equal to one, and C was the concentration of probe molecules in the solution (unit: mol cm^−3^). The estimated ECSA for PaperLIG was 0.17 cm^2^ and this value was 1.9 times larger than its geometric area (0.09 cm^2^), which was similar to the previously reported ECSA for PILIG [43].

The EIS data of the PaperLIG electrode in the PBS solution containing 5 mM K_3_Fe(CN)_6_/K_4_Fe(CN)_6_ with 0.1 M KCl were also measured (Figure 3d). As illustrated in Figure 3d, two semicircles appeared in the EIS of PaperLIG, and thus the simply modified Randles circuit model was not applicable for describing the impedimetric response of PaperLIG. As indicated by the SEM observation (Figure 2e–h), the porosity of the PaperLIG is contributed by two parts: the inter-fiber structure and the intra-fiber structure [44]. The high porosity of the intra-fiber structure generates an additional interfacial layer at the PaperLIG/electrolyte solution interface [44]. In order to simulate this additional interfacial layer, a parallel resistor and capacitor were added, which were also employed to characterize the impedimetric behaviors of porous electrodes for batteries [45,46]. As shown in Figure 3d, an equivalent circuit was used to fit the EIS data, which includes a solution resistance (*R_S_*), an interfacial layer resistance (*R_SEI_*), an interfacial layer capacitance (*CPE_SEI_*), a double layer capacitance (*CPE_dl_*) in parallel a charger transfer resistance (*R_ct_*), and a Warburg resistance (*W*). Based on the fitting result, the *R_ct_* of PaperLIG in Figure 3d is ~120.92 ± 9.88 Ω (area: 3 × 3 mm^2^) (*n* = 5), which was comparable with previously reported LIG-based biosensing electrodes [17,47]. Therefore, the results obtained from CV and EIS suggest that the fabricated PaperLIG electrode is suitable for electrochemical sensing purposes.

### 3.3. Development of PaperLIG-Based Glucose Biosensor

After confirming the electrochemical performance of the PaperLIG electrode, PaperLIG was used to develop a glucose biosensor. Firstly, aminoferrocene (AFc) was drop-cast on the PaperLIG electrode as the mediator layer, and glucose oxidase was then drop-cast on the PaperLIG/AFc to selectively react with the glucose molecules in the sample [1,28,48]. Finally, a Nafion thin film was applied to avoid the loss of enzyme from the PaperLIG electrode and the possible interference from other redox-reactive species [28,29,48,49]. As a reference, the CV of the PaperLIG electrode in the PBS solution containing 1 mg/mL AFc is shown in Figure S4b. The CV peak current showed great linearity with the square root of the scan rate (Figure S4c), which indicates that the mass-transfer process of solved AFc to the PaperLIG electrode is mainly diffusion-controlled [38]. The CV of the PaperLIG/AFc/GOx/Nafion electrode in the PBS solution with different scan rates (1–250 mV/s) are illustrated in Figure S4d. Similar to the results shown in Figure S4c, the peak CV current density of adsorbed AFc on the PaperLIG/AFc/GOx/Nafion electrode shows good linearity with the square root of the scan rate (Figure S4e). The poorer linearity for plots of the peak current density versus the scan rate could be noticed in Figure S4f. The great linearity of the plots in Figure S4e implies that the redox reaction of AFc with the Nafion film of the PaperLIG/AFc/GOx/Nafion electrode is diffusion-controlled [38], similar to the previously reported Nafion coated glucose biosensor and other Nafion coated electrodes [28,50]. 

The glucose-sensing mechanism of the PaperLIG/AFc/GOx/Nafion electrode is illustrated on the right in Figure 1 and the supporting information (Sensing mechanism for the PaperLIG-based glucose biosensor). After the fabrication of PaperLIG/AFc/GOx/Nafion, the electrode was placed in the PBS for stabilization for at least 2 h before use. Then, the OCP (open circuit potential) of the developed biosensor was measured against a glass Ag/AgCl reference electrode. It should be noted that the drop-cast mediator layer was composed of both reduced form (AFc^+^) and oxidized form (AFc), and the purchased aminoferrocene powder contained a higher amount of reduced form. Therefore, as explained by Equation (S5), the OCP of the biosensor was closer to the reduction potential of aminoferrocene and the measured OCP value was around −100 mV. Finally, the chronoamperometric method was used with the applied potential (−90 mV, 10 mV higher than the measured OCP) to detect the glucose concentration in the N_2_-saturated PBS and artificial saliva solutions with different glucose concentrations.

**Figure 4:**
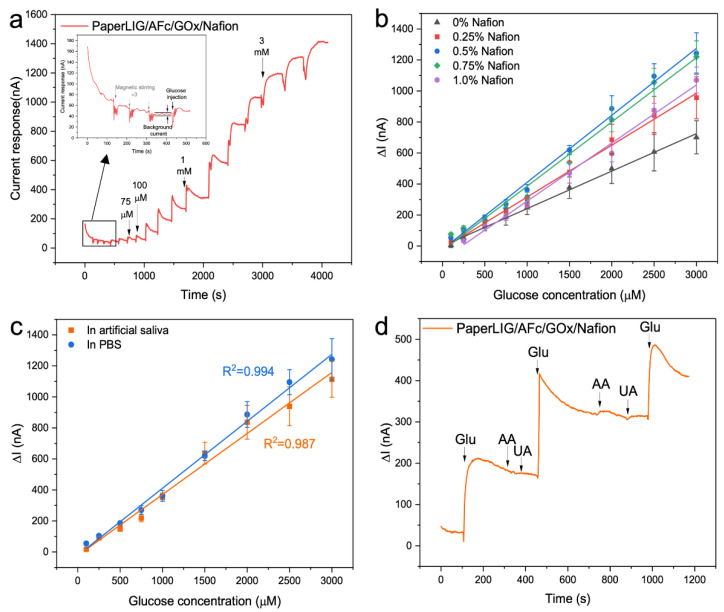
(**a**) Amperometric response of PaperLIG/AFc/GOx/Nafion electrode to different glucose concentrations in the PBS (applied potential for amperometry: −90 mV, Nafion concentration 0.25%). The inset shows the enlarged response from the black square marked area. (**b**) The corresponding calibration curves for PaperLIG-based glucose biosensor in the PBS were prepared using different concentrations of Nafion (*n* = 3). (**c**) The corresponding calibration curves for PaperLIG/AFc/GOx/Nafion (0.5%) glucose biosensor in artificial saliva (orange line) and the PBS (blue line) (*n* = 3). (**d**) Interference test of the PaperLIG-based glucose biosensor: response to 500 µM glucose in the presence of common interfering substances in artificial saliva (20 µM AA and 200 µM UA).

The amperometric approach was used to evaluate the sensing performance of the PaperLIG-based glucose biosensor, as illustrated in Figure 4a. As shown in the inset of Figure 4a, prior to the injection of glucose, the solution was magnetically stirred three times. It was noted that magnetic stirring generated the fluctuation of the current signal, but no current increment was observed, indicating that magnetic stirring did not cause the current response of the biosensor. As Figure 4a shows, the current signal increases after adding glucose into the solution, and this applied potential (−90 mV) is much lower than that of most developed LIG-based glucose biosensors (Table S2), which can avoid the oxidation of possible redox-active interfering substances in the saliva sample [29]. The Nafion concentration for this first biosensor was 0.25%, and the biosensor showed a linear range between 0.1 and 3 mM for glucose levels in the PBS with good linearity (R^2^ = 0.995) (Figure 4b). The LOD was determined as the mean background + 3 SDs of the background [51], and the LOD was around 50–75 µM for this glucose biosensor. As a comparison, the PaperLIG/GOx/Nafion (0.5%) biosensor was also prepared and measured in the PBS using the amperometric method under −90 mV (Figure S5a). Previously, it was reported that graphene material can facilitate the DET (direct electron transfer) of GOx, which might be employed to develop the third generation glucose biosensor [52,53,54]. As the co-enzyme of GOx, it was reported that the redox potential for FAD on the LIG electrode is around −485 mV [54]. As demonstrated in Figure S5, no obvious current response could be observed, which indicated that under applied potential (−90 mV) the direct electron transfer from the enzyme to the electrode was not feasible and the usage of a mediator was necessary.

Then, the effect of the Nafion concentration on the performance of glucose sensing was also investigated, and Nafion solutions of different concentrations (0.25–1%) were prepared for biosensor construction. As a reference, PaperLIG/AFc/GOx biosensors without a Nafion thin film were also fabricated. As Figure 4b shows (black line), without the protective layer, the fabricated PaperLIG/AFc/GOx biosensors showed the lowest current signal and poorest sensitivity, which might be due to the leaching of enzyme and mediator from the surface of the PaperLIG electrode. As shown in Figure 4b and Table 1, with increasing concentration of the Nafion casting solution, the sensitivity of the glucose biosensor firstly increases to the highest value for a concentration of 5%, which can be attributed to the improved immobilization of the mediator and enzyme with a thicker Nafion thin film. Nevertheless, the glucose biosensors prepared using higher Nafion concentrations (>0.5%) showed poorer sensitivity. Meanwhile, it can be noticed in Table 1 that with too high a concentration of Nafion on the biosensor, the limit of detection (LOD) and linear sensing range of the biosensor are affected. Without a Nafion thin film, the PaperLIG/AFc/GOx biosensor shows the lowest LOD (25–50 µM), and for Nafion concentrations ranging from 0.25% to 0.75%, the developed biosensors present a similar LOD and linear sensing range. However, the PaperLIG-based biosensor prepared using the 1% Nafion solution shows a poorer LOD and linear sensing range (Table 1). Such results are in agreement with other previously reported electrochemical biosensors using Nafion thin films [55,56,57]. The observed effect of a high-concentration (>0.5%) Nafion casting solution on the performance of the developed glucose biosensor could be attributed to the thickness of the Nafion film [55,56,57]. For the same electrode area, as the Nafion film becomes thicker, the diffusion of glucose molecules might be hindered, which accounts for the poorer performance of the biosensors prepared using more concentrated Nafion solutions. 

An SEM observation was used to investigate the effect of the concentration of casted Nafion solution on the thickness of the Nafion film on PaperLIG (Figure S6). Due to the highly porous and fibrous structure of the PaperLIG, instead of forming a flat film structure, the Nafion film is formed on the surface of each PaperLIG fiber (Figure S6), making it difficult to determine the exact thickness of the Nafion film on the PaperLIG using the cross-sectional SEM (Figure S6c,f,i,l). From the top-view SEM of the PaperLIG/Nafion, however, the influence of the casted Nafion concentration could still be observed. For the PaperLIG/Nafion (0.25%), the edge structure of the graphene flake could be observed clearly (Figure S6a,b). For the PaperLIG/Nafion (0.5%), the Nafion thin film covered the majority of the graphene flake’s edge structure (Figure S6d,e). With a concentration increase from 0.5% to 1%, the surface of PaperLIG’s fibrous structure was coated with a thicker Nafion film, and excess Nafion began to aggregate on the surface of PaperLIG (Figure S6g,h,j,k). Especially for the PaperLIG/Nafion (1%), the cross-sectional SEM (Figure S6l) revealed the Nafion layer on the PaperLIG. As a reference, the flat Nafion film on polyimide with varying casted concentration (0.25–1%) was prepared using the same drop-casting method, and its cross-sectional SEM was acquired (Figure S7). The Nafion film thickness grew from 4.6–8.9 µm to 11.3–21.5 µm as the cased Nafion concentration rose from 0.25% to 1% (Figure S7). Based on the SEM observation, it could be determined that for the same surface area of the substrate, a higher concentration of the casted Nafion solution could produce a thicker Nafion film, and that such a thicker and aggregated Nafion film structure may hinder the diffusion of glucose molecules. Based on the experimental results from Figure 4b, we determined that the optimized Nafion concentration for our PaperLIG-based biosensor is 0.5%. Nevertheless, the use of different methods for preparing and drying the Nafion film can also affect the performance of the Nafion-coated biosensor [58,59]. In this study, the Nafion thin film was prepared by drying under ambient conditions after simple drop-casting on the electrode. Thus, it should be noted that different Nafion coating methods as well as different kinds of LIG material may result in a different relationship between the biosensing performance and the Nafion concentration. 

For the PaperLIG/AFc/GOx/Nafion (0.5%), the catalytic efficiency of the enzymes was investigated through the apparent Michaelis–Menten constant ($K_{m}^{app}$) by using the Lineweaver–Burke equation (Equation (S3)) (Figure S5b) [27,60]. The $K_{m}^{app}$ was estimated to be 3.13 mM which was similar to previous reported LIG-based glucose biosensors ($K_{m}^{app}=3.75\;\mathrm{mM}$) [27] and ferrocene mediated glucose biosensor ($K_{m}^{app}=10.36\;\mathrm{mM}$) [61]. Figure 4c illustrates the calibration curve for evaluating the sensing performance of the PaperLIG/AFc/GOx/Nafion (0.5%) in artificial saliva. The PaperLIG/AFc/GOx/Nafion (0.5%) biosensor exhibits the same LOD (50–75 µM) and linear range in artificial saliva (0.1–3 mM) as in the PBS. As shown in Figure 4c, a lower sensitivity of the PaperLIG biosensor in artificial saliva can be observed, which may be owing to the different pH and ionic strength [62,63]. Ascorbic acid (AA) and uric acid (UA) were also examined as possible interfering compounds in saliva samples. The interference experiment for the biosensor was carried out in artificial saliva using a PaperLIG-based glucose biosensor containing 0.5% Nafion and the results are shown in Figure 4d. 20 µM AA and 200 µM UA (the basal concentrations of these compounds in saliva [51]) did not cause any detectable current change in the artificial saliva with a 500 µM glucose concentration, and a further increase in the glucose concentration caused a rapid response (Figure 4d). The low applied potential (−90 mV) for the measurement and the application of the Nafion thin film prevents possible interference from redox-reactive species in saliva [29].

## 4. Discussion

Previously, researchers developed the Fc-based glucose biosensor using a similar drop-casting method, with the mediator density and enzyme density for GCE (geometric area: 0.12 cm^2^) being 0.16–0.78 mg/cm^2^ and 0.08–0.42 mg/cm^2^, and the mediator/enzyme density ratio being 1.86–2 [28]. The as-prepared Fc-based glucose biosensor shows a higher linear sensing range of up to 16 mM [28]. Considering the poorer conductivity and the large surface area of PaperLIG, a higher mediator density (AFc: 1.12 mg/cm^2^) and enzyme density (GOx: 1.11 mg/cm^2^) were applied for the PaperLIG electrode (geometric area: 0.09 cm^2^) in this study for improving the electron transfer efficiency. In this study, the mediator/enzyme density ratio in this study was ~1, and the developed PaperLIG-based glucose biosensors showed a linear response from 0.1–3 mM. Considering the much higher linear sensing range of the glucose biosensor prepared by previous researcher with a higher mediator/enzyme ratio, the applied mediator/enzyme ratio in this study may not be the optimal ratio for the glucose biosensing purpose. Further investigation of this mediator/enzyme density ratio may improve the sensing performance of the glucose biosensing, which will be the next step of this study. 

As summarized in Table 2, compared with other reported non-invasive glucose biosensors, the developed PaperLIG-based glucose biosensor can be operated at a lower applied potential, which is beneficial for reducing possible interference from other redox-active species. As shown in Table 2, the sensing performance of the PaperLIG-based glucose biosensor shows sensing performance comparable to that reported in previous studies. In addition, the sensing performance can be improved by conducting additional research into the optimal mediator and enzyme concentrations. More importantly, the proposed PaperLIG-based biosensor can be fabricated at a low cost and disposed of simply. Despite the fact that compared to first generation glucose biosensors, the second generation glucose biosensors do not require the involvement of oxygen and can be operated at a lower potential, there are still drawbacks. To construct the second generation glucose biosensor, an artificial mediator is required, and the majority of synthetic mediators are harmful to humans, necessitating additional precautions when constructing the wearable or implantable second generation glucose biosensor [1].

As a demonstration of the potential of the PaperLIG-based glucose biosensor for future healthcare applications, the 3-PaperLIG sensing device was fabricated successfully (Figure S8a). As the figure shows, the 3-PaperLIG sensing device is composed of a PaperLIG-based working electrode (WE), a PaperLIG-based counter electrode (CE), and an Ag/AgCl ink-based reference electrode (RE). In order to control the 3-PaperLIG sensing device, a miniaturized analysis system (miniAS) (Figure S8b) was designed and developed. The compactable size (58.7 × 52.2 × 23.3 mm^3^) and light-weight (~46 g) of the miniAS demonstrate its potential for wearable or portable healthcare applications. By connecting the 3-PaperLIG sensing device with the miniAS, cyclic voltammetry measurements under different scan rates could be performed (Figure S8c,d). After modifying the WE with the biorecognition element, the 3-PaperLIG device is capable of detecting glucose or other molecules, which will be the next step in this study. The fabrication process for the 3-PaperLIG device and the developing process for the miniAS can be found in the supporting information. Thus, based on the experimental results, we believe that our developed PaperLIG-based glucose biosensor shows great promise for the future of healthcare management. 

## 5. Conclusions

In this research, the development of a disposable glucose biosensor using the PaperLIG material is introduced. After investigation and optimization of the speed of the second laser engraving step, highly conductive PaperLIG material was obtained (sheet resistance: 38.64 ± 2.11 Ω·sq^−1^). Using SEM, high porosity and a graphene flake structure were observed on each fiber of the PaperLIG material, which is totally different from the structures of the pristine paper and the PaperAC material. The high porosity and large real surface area of PaperLIG are favorable for immobilizing a mediator and enzyme. Electrochemical measurements based on CV and EIS were applied for the electrochemical characterization of the PaperLIG material. To fit the EIS data, additional capacitor and resistor elements were introduced into the equivalent circuit to model the interfacial layer caused by the highly porous intra-fiber structure of PaperLIG, which is different from most reported LIG-based electrodes. Electrochemical measurements confirmed that PaperLIG is suitable for application in electrochemical biosensors. By drop-casting aminoferrocene, glucose oxidase, and Nafion on the surface of the PaperLIG, a glucose biosensor was successfully fabricated, which showed a good response to glucose concentrations in PBS ranging from 100 µM to 3000 µM with good linearity (R^2^ = 0.996). The effect of the concentration of the Nafion casting solution on the sensing performance of the glucose biosensor was also investigated, and the results showed that a 0.5% concentration led to better sensing performance among the five Nafion concentrations tested. Ascorbic acid and uric acid were not found to interfere with the glucose measurements. Moreover, successful electrochemical measurement using the 3-PaperLIG device and miniaturized analysis system demonstrates that the PaperLIG-based biosensor is capable of being used in a wearable or portable device. Based on the experimental results, as a disposable biosensor that can be gasified simply, the developed PaperLIG-based biosensor shows great potential for application in future healthcare monitoring devices.

## Figures and Tables

**Table 1:** The performance of PaperLIG-based glucose biosensor prepared with various Nafion solution concentrations.

Nafion Concentration (%)	LOD (µM)	Sensitivity (nA/µM)	Linear Range (mM)	R^2^
0	25–50	0.242	0.1–3	0.997
0.25	50–75	0.335	0.1–3	0.996
0.5	50–75	0.432	0.1–3	0.994
0.75	50–75	0.415	0.1–3	0.988
1	200–250	0.364	0.25–3	0.985

**Table 2:** The comparison of PaperLIG-based glucose biosensors with other kinds of glucose biosensors. A great number of + signs indicate a higher evaluation for the corresponding parameter of the biosensor.

Glucose Biosensor	Applied Potential (V)	LOD (µM)	Linear Range (mM)	Manufacturing Simplicity	Manufacturing Cost	Disposability *	Ref.
PET */Au/PB */GOx	+0.1 V	2.7	0.02–1.11	+	+	++	[64]
PET/SPCE */PB/GOx/Nafion	−0.2 V	40	0.1–1.4	+++	++	++	[51]
PILIG/Cu NPs	+0.5 V	0.39	1–6.0	++	+++	+	[65]
PILIG/Pt NPs/PPD/GOx	+0.6 V	8	0.008–1	++	+++	+	[62]
PILIG/PB/Chitosan/GOx	−0.05 V	13.7 ± 0.5	<1.5	++	+++	+	[21]
PaperLIG/AFc/GOx/Nafion	−0.09 V	50–75	0.1–3	+++	+++	+++	This work

* Disposability: The property of being disposable in a simple and low-cost way after using; PET: polyethylene terephthalate; PB: prussian blue; SPCE: screen-printed carbon paste electrode; Cu NPs: copper nanoparticles; Pt NPs: platinum nanoparticles; PPD: polyphenylenediamine.

## Data Availability

The data presented in this work are not publicly available at this time but can be obtained upon reasonable request from the authors.

## Supplementary Materials

The following supporting information can be downloaded at: https://www.mdpi.com/article/10.3390/bios12110995/s1, Equations (S1)–(S5): Sensing mechanism for the PaperLIG-based glucose biosensor; Preparation of Nafion-coated polyimide film; Fabrication of 3-PaperLIG device; Development of miniaturized analyzing system; Table S1. Element composition of EDS analysis of FP (filter paper), FP with FR (filter paper with fire-retardant), PaperAC, and PaperLIG. Table S2 Comparison of the applied potential for glucose biosensor based on the LIG derived from polyimide. Pt NPs: platinum nanoparticle; Cu NPs: copper nanoparticle; PB: Prussian blue. Figure S1: Raman spectrum of PaperAC; Figure S2: EDS analysis of filter paper, filter paper with fire retardant, PaperAC and PaperLIG; Figure S3: SEM observation for PaperAC and PaperLIG; Figure S4: Cyclic voltammetry for PaperLIG electrode and PaperLIG/AFc/GOx/Nafion electrode; Figure S5: Amperometric response of PaperLIG/GOx/Nafion biosensor and the kinetic analysis of PaperLIG/AFc/GOx/Nafion biosensor; Figure S6: SEM observation of PaperLIG/Nafion material; Figure S7: SEM observation of Polyimide/Nafion material; Figure S8: Illustration of electrochemical measurement using the 3-PaperLIG device and the miniAS. Refs. [66,67,68,69,70,71] are cited in supplementary materials.
