# Supplementary material for: A Mediated Enzymatic Electrochemical Sensor Using Paper-Based Laser-Induced Graphene
Source: Biosensors (Basel). 2022 Nov 9;12(11):995. doi: 10.3390/bios12110995 (PMC9688852; doi:10.3390/bios12110995)
Supplement: Supplementary file 1 [file biosensors-12-00995-s001.zip › biosensors-1978351-supplementary.pdf]

Supplementary Materials

A mediated enzymatic electrochemical sensor using paper-based laser-induced graphene

Panpan Gao [1], Toshihiro Kasama [1] Jungchan Shin [1], Yixuan Huang [2] and Ryo Miyake[1,*]

[1] Microfluidic Integrated Circuits Research Laboratory, Bioengineering, School of Engineering, The University of Tokyo
[2] Bioengineering, School of Engineering, The University of Tokyo
* Correspondence: trmiyake@mail.ecc.u-tokyo.ac.jp; Tel.: +81-044-223-7032

Equation S1

$$E = P \times t = P \times \frac{S}{V} \tag{S1}$$

where E is the fluence of the laser (unit: J), P is the laser power (unit: W), t is the engraving time, S is the area of the engraved pattern, and V is the laser engraving speed (unit: inch/s).

Equation S2

$$I_p = 2.69 \times 10^5 A D^{1/2} n^{3/2} \nu^{1/2} C \tag{S2}$$

Where I_p represents the voltametric peak current; A represents the electrode's active surface area (unit: cm^2); D represents the diffusion coefficient of the redox probe (unit: $cm^2{*}s^{-1}$); n represents the number of electrons transferred during the redox reaction (the number of transferred electrons for $[Fe(CN)6]^{3-/4-}$ is 1.0); ν represents the scan rate (unit: $V{*}s^{-1}$); and C represents the concentration of the redox probe (unit: $mol{*}cm^{-3}$).

Equation S3

$$\frac{1}{I} = \frac{K_m^{app}}{I_{max}} \frac{1}{C_{substrate}} + \frac{1}{I_{max}} \tag{S3}$$

where I represents the current, I_{max} represents the maximum current measured under saturated substrate conditions, and $C_{substrate}$ is the glucose concentration.

Sensing mechanism for the PaperLIG-based glucose biosensor

The glucose molecule is oxidized by GOx (FAD) and produces D-glucono-δ lactone, and simultaneously GOx (FAD) is reduced to GOX (FADH). Then, AFc^+ (aminoferrocenium, the oxidized form of AFc) is reduced by GOx (FADH) and transforms into AFc and, meanwhile, GOx (FADH) is oxidized to GOx (FAD). When applying an appropriate potential to the electrode, AFc is oxidized to AFc^+ and transfers one electron to the electrode, which results in a current response, and this current signal can be used to indicate the glucose concentration [1–3]. To detect the concentration of aminoferrocene, the chronoamperometric method was used to oxidize the aminoferrocene at a potential close to the open circuit potential (OCP). The OCP of the electrochemical cell can be described by the Nernst equation (S4) [4].

$$E = E^0 + \frac{RT}{nF} \ln \frac{(Ox)}{(Red)} \tag{S4}$$

where $E$is the potential of the electrochemical cell; the E^0 is the standard potential of a redox species; R is the universal gas constant; T is temperature; n is the number of electrons; F is Faraday's constant; and Ox and Red are the relative activities of the oxidized form and reduced form of the analyte in the electrochemical system.

After replacing the E^0 with the formal potential $E^{0\prime}$ and n is set to be 1, Equation (S4) is transformed to Equation (S5), which indicates that the OCP of the developed PaperLIG biosensor is strongly governed by the concentration ratio of AFc^+/AFc [4].

$$E = E^{0\prime} + \frac{RT}{F}\ln\frac{[AFc^+]}{[AFc]} \quad (S5)$$

An increase in the glucose concentration can promote the production of AFc and cause a negative shift in the OCP of the PaperLIG biosensor. Therefore, an applied potential slightly more positive than the original OCP of the biosensor can drive the oxidation reaction to occur. The increasing glucose concentration can stimulate an increasing oxidation current for AFc, which can be used to indicate the glucose concentration. The mechanism of the measurement is similar to that of a previously reported biosensor based on amperometry at OCP [5] and other kinds of OCP-based biosensors [6–8].

Preparation of Nafion-coated polyimide film

The square polyimide (PI) film (geometric area: 3 × 3 mm^2) was cut by a CO_2 laser system. After cleaning the cut PI sample, 4 µL of 0.25%-1.0% Nafion solution was drop-casted on the dried PI sample followed by drying under ambient conditions for 1 h. Finally, the Nafion-coated PI samples were observed by the scanning electron microscope.

Fabrication of 3-PaperLIG device

The pattern of the 3-PaperLIG device was designed using CorelDRAW software. After pretreatment of paper with fire retardant, filer paper was then engraved by the CO_2 laser system with the same setting of PaperLIG electrode. Copper tape (Teraoka Seisakusho, Japan) was used as the connection pad for the 3-PaperLIG device, and silver paste was used to build the connection between PaperLIG and copper tape. The reference electrode was prepared by casting the Ag/AgCl ink (BAS, Japan) on the RE part for 3-PaperLIG, and then the ink was curried in a 120 °C drying oven for 15 mins. After that, epoxy glue was coated on the silver paste and the backside of the filter paper to serve as an insulating barrier to avoid direct contact between the samples and the silver paste.

Development of a miniaturized analyzing system (miniAS)

A miniaturized analyzing system (miniAS) was developed for controlling the 3-PaperLIG device. The miniAS consists of a chip-based potentiostat (Emstat Pico All, PalmSens, Netherlands), a Bluetooth model (HC06), an Arduino mini pro board (Arduino, Italy), a 3.7V Lipo battery (EEMB, China), and a 3D-printed outer case. The 3D printed case was designed by Fusion 360 (Autodesk, USA) and printed using a 3D printer (EDEN260VS, Stratasys, USA).

Table S1. Element composition of EDS analysis of FP (filter paper), FP with FR (filter paper with fire-retardant), PaperAC, and PaperLIG.

Atomic (%)	FP	FP with FR	PaperAC	PaperLIG
C	52.54±0.31	45.52±0.51	68.24±0.54	80.27±0.27
N	0	9.54±0.36	7.79±0.85	5.79±0.81
O	47.46±0.31	42.70±0.94	21.58±0.71	11.53±0.88
P	0	1.29±0.38	2.15±0.18	2.20±0.19

S	0	0.95±0.05	0.28±0.06	0.22±0.01

Table S2 Comparison of the applied potential for glucose biosensor based on the LIG derived from polyimide. Pt NPs: platinum nanoparticle; Cu NPs: copper nanoparticle; PB: Prussian blue**.**

Biosensor structure	Applied potential	Ref.
PILIG/Pt NPs/GOx	+0.4 V	[9]
PILIG/Pt NPs/GOx/Nafion	+0.6 V	[10]
PILIG/GOx/Chitosan	+0.8 V	[11]
PILIG/Cu NPs	+0.5 V	[12]
PILIG/Cu NPs	+0.45 V	[13]
PILIG/PB/Chitosan-GOx	-0.05 V	[14]
PaperLIG/AFc/GOx/Nafion	-0.09 V	This work

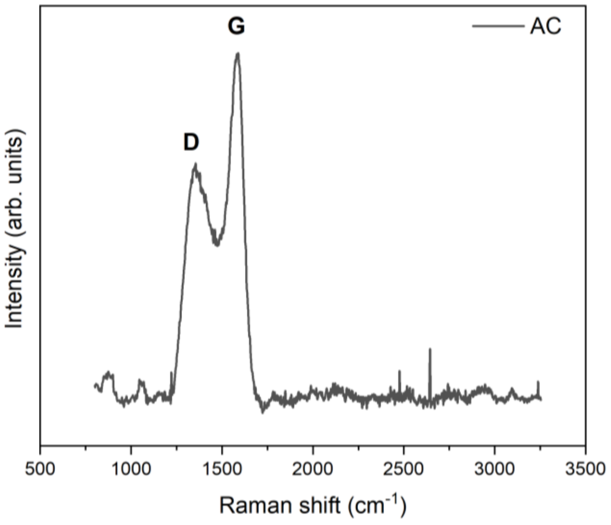

Figure S1. Raman spectrum of PaperAC after first laser engraving of paper material.

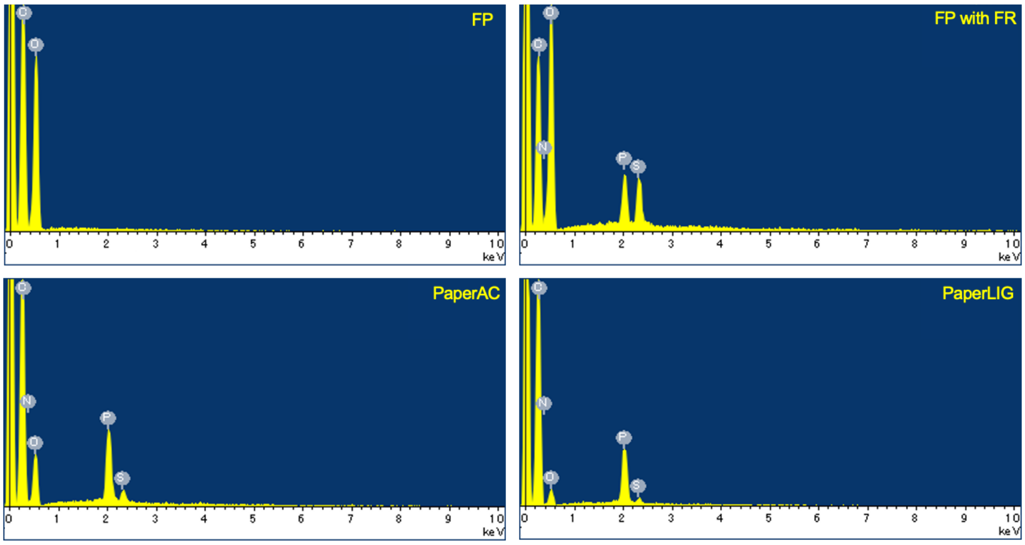

Figure S2. EDS analysis of FP (filter paper), FP with FR (filter paper with fire-retardant), PaperAC (Paper-based amorphous carbon), and PaperLIG (Paper-based laser-induced graphene).

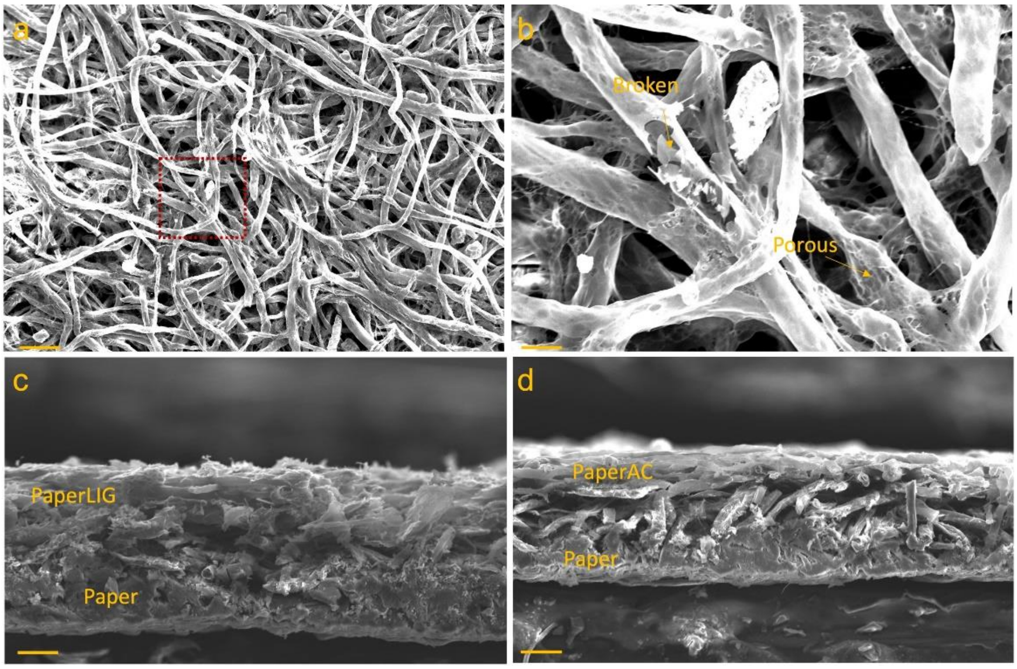

Figure S3. SEM observation for PaperAC and PaperLIG. (a) SEM image of PaperAC. Scale: 100 µm. (b) Magnified SEM of the red rectangular part of Fig. S3a. Scale: 20 µm. (c) Cross-sectional SEM of PaperLIG. Scale: 50 µm. (d) Cross-sectional SEM of PaperAC. Scale: 66.6 µm.

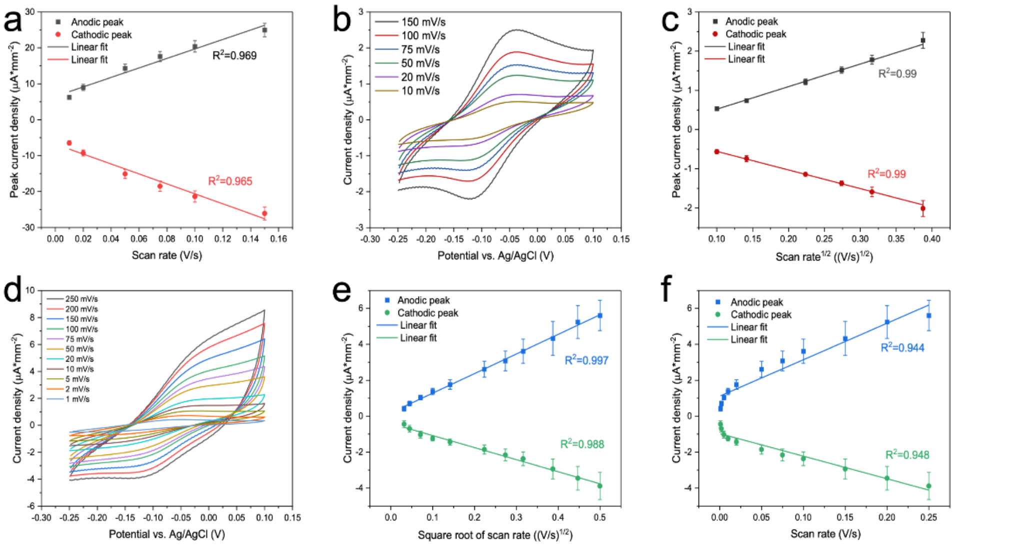

Figure S4. Cyclic voltammetry for PaperLIG electrode and PaperLIG/AFc/GOx/Nafion electrode. (a) The plot of peak current density (n=10) versus the scan rate for the CV of PaperLIG electrodes in the PBS contained $K_3Fe(CN)_6/K_4Fe(CN)_6$ (Figure 3b). (b) CV of PaperLIG electrode in the PBS solution containing 1 mg/ml AFc under different scan rates (10-150 mV/s). (c) The plot of peak current density (n=5) versus the square root of scan rate for the CV of PaperLIG electrode in the PBS solution containing 1mg/ml AFc. (d) CV of PaperLIG/AFc/GOx/Nafion electrode in the PBS solution under different scan rates (1-250 mV/s). (e) The plot of peak current density (n=5) versus the square root of scan rate for the CV of PaperLIG/AFc/GOx/Nafion electrode in the PBS solution. (f) The plot of peak current density (n=5) versus the square root of scan rate for the CV of PaperLIG/AFc/GOx/Nafion electrode in the PBS solution.

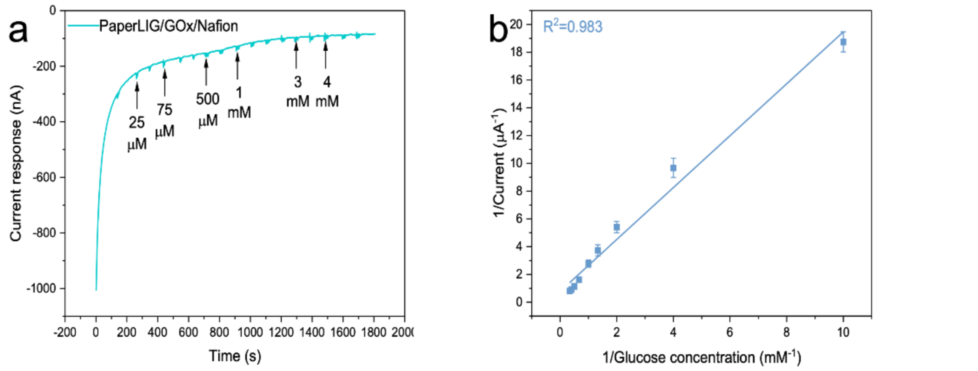

Figure S5. Amperometry of PaperLIG/GOx/Nafion biosensor and the kinetic analysis of PaperLIG/AFc/GOx/Nafion biosensor. (a) The amperometric response of PaperLIG/GOx/Nafion biosensor in N_2 saturated PBS (applied potential: -90 mV). (b) The Lineweaver-Burk plot of the PaperLIG/AFc/GOx/Nafion (0.5%) glucose biosensor derived from the calibration curve in Figure 4b.

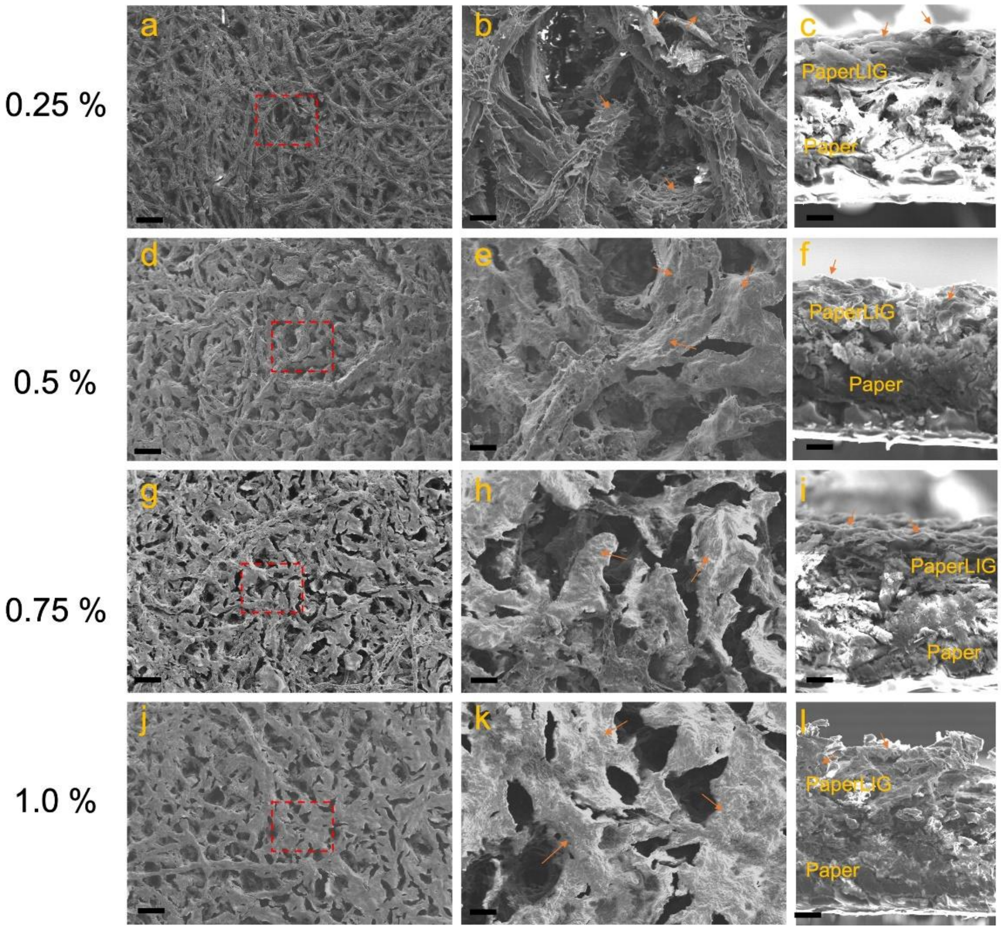

Figure S6. SEM observation of PaperLIG/Nafion material. (a), (d), (g), and (j). The top-view SEM observation of PaperLIG/Nafion material with different casted Nafion concentrations (0.25%-1%). Scale: 100 μm. (b), (e), (h), and (k). The amplified SEM image of the red rectangular part of Figure S5a, S5d, S5g, and S5j, respectively. The orange arrow is indicating the coated Nafion polymer. Scale: 20 μm. (c), (f), (i), and (l). The cross-sectional SEM of PaperLIG/Nafion material with different casted Nafion concentrations (0.25%-1%). The orange arrow is indicating the coated Nafion polymer. Scale: 33.3 μm.

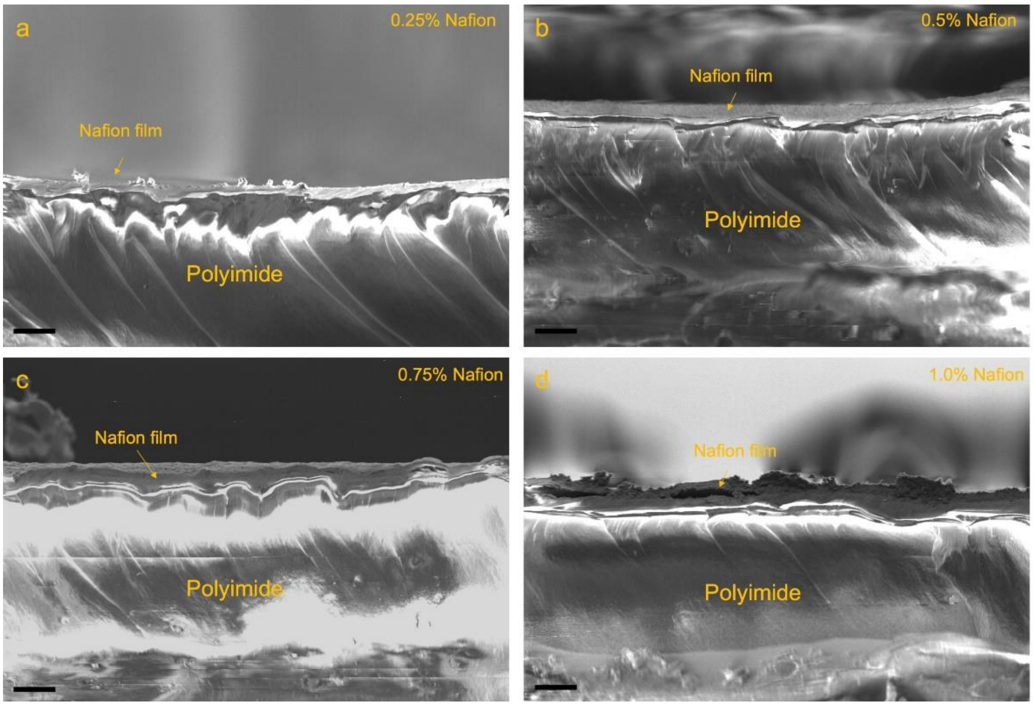

Figure S7. SEM observation of Polyimide/Nafion material. The cross-sectional SEM observation of polyimide/Nafion material with different casted Nafion concentrations (0.25%-1%). Scale: 20 μm.

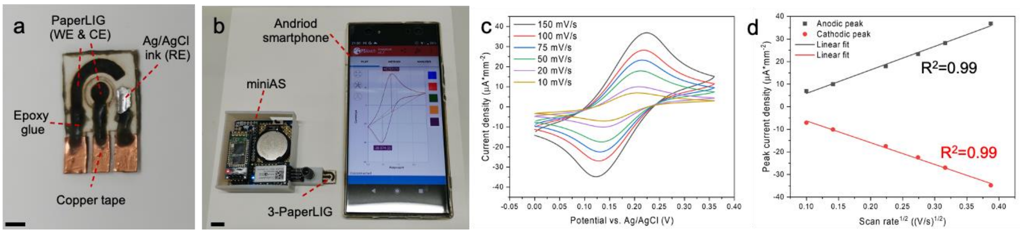

Figure S8. Illustration of electrochemical measurement using 3-PaperLIG device and miniAS. (a) The fabricated 3-PaperLIG device, which composed of a PaperLIG WE, a PaperLIG CE, and an Ag/AgCl ink RE. Scale: 3 mm. (b) The 3-PaperLIG device is connected with a developed miniaturized analyzing system (miniAS). Scale: 10 mm. (c) Cyclic voltammetry of 3-PaperLIG electrode in PBS solution containing 5 mM $K_3Fe(CN)_6/K_4Fe(CN)_6$ with 0.1 M KCl under different scan rates (10-150 mV/s) controlled by miniAS. (d) The plot of peak current density versus the square root of the scan rate for the CV of 3-PaperLIG electrodes (Figure S8c).

Reference

1. Teymourian, H.; Barfidokht, A.; Wang, J. Electrochemical Glucose Sensors in Diabetes Management: An Updated Review (2010–2020). *Chem. Soc. Rev.* **2020**, *49*, 7671–7709.

2. Vaillancourt, M.; Wei Chen, J.; Fortier, G.; Bélanger, D. Electrochemical and Enzymatic Studies of Electron Transfer Mediation by Ferrocene Derivatives with Nafion-Glucose Oxidase Electrodes. *Electroanalysis* **1999**, *11*, 23–31.

3. Fiorito, P.A., Torresi, S.I.C. Glucose Amperometric Biosensor Based on the Co-Immobilization of Glucose Oxidase (GOx) and Ferrocene in Poly(Pyrrole) Generated from Ethanol / Water Mixtures. *J. Braz. Chem. Soc.* **2001**, *12*, 729-733. https://doi.org/10.1590/S0103-50532001000600007

4. Elgrishi, N.; Rountree, K.J.; McCarthy, B.D.; Rountree, E.S.; Eisenhart, T.T.; Dempsey, J.L. A Practical Beginner's Guide to Cyclic Voltammetry. *J Chem Educ* **2018**, 10.

5. Brânzoi, F.; Brânzoi, V. *Amperometric Urea Biosensor Based Metallic Substrate Modified with a Nancomposite Film*; IntechOpen: Rijeka, Croatia, **2013**; ISBN 978-953-51-1035-4.

6. Su, D.; Feng, B.; Xu, P.; Zeng, Q.; Shan, B.; Song, Y. Covalent Organic Frameworks and Electron Mediator-Based Open Circuit Potential Biosensor for *in Vivo* Electrochemical Measurements. *Anal. Methods* **2018**, *10*, 4320–4328. https://doi.org/10.1039/C8AY01386A.

7. Song, Y.; Su, D.; Shen, Y.; Liu, H.; Wang, L. Design and Preparation of Open Circuit Potential Biosensor for in Vitro and in Vivo Glucose Monitoring. *Anal. Bioanal. Chem.* **2017**, *409*, 161–168. https://link.springer.com/article/10.1007/s00216-016-9982-1.

8. Charoenkitamorn, K.; Tue, P.T.; Kawai, K.; Chailapakul, O.; Takamura, Y. Electrochemical Immunoassay Using Open Circuit Potential Detection Labeled by Platinum Nanoparticles. *Sensors* **2018**, *18*, 444. https://doi.org/10.3390/s18020444.

9. Yoon, H.; Nah, J.; Kim, H.; Ko, S.; Sharifuzzaman, M.; Barman, S.C.; Xuan, X.; Kim, J.; Park, J.Y. A Chemically Modified Laser-Induced Porous Graphene Based Flexible and Ultrasensitive Electrochemical Biosensor for Sweat Glucose Detection. *Sens. Actuators B Chem.* **2020**, *311*, 127866. https://doi.org/10.1016/j.snb.2020.127866.

10. Lu, Z.; Wu, L.; Dai, X.; Wang, Y.; Sun, M.; Zhou, C.; Du, H.; Rao, H. Novel Flexible Bifunctional Amperometric Biosensor Based on Laser Engraved Porous Graphene Array Electrodes: Highly Sensitive Electrochemical Determination of Hydrogen Peroxide and Glucose. *J. Hazard. Mater.* **2021**, *402*, 123774.

11. Settu, K.; Chiu, P.-T.; Huang, Y.-M. Laser-Induced Graphene-Based Enzymatic Biosensor for Glucose Detection. *Polymers* **2021**, *13*, 2795.

12. Zhang, Y.; Li, N.; Xiang, Y.; Wang, D.; Zhang, P.; Wang, Y.; Lu, S.; Xu, R.; Zhao, J. A Flexible Non-Enzymatic Glucose Sensor Based on Copper Nanoparticles Anchored on Laser-Induced Graphene. *Carbon* **2020**, *156*, 506–513. https://doi.org/10.1016/j.carbon.2019.10.006

13. Juska, V.B.; Juska, G. Copper-Nanostructure-Modified Laser-Scribed Electrodes Based on Graphitic Carbon for Electrochemical Detection of Dopamine and Glucose. *J. Chem. Technol. Biotechnol.* **2021**, *96*, 1086–1095. https://doi.org/10.1002/jctb.6620.

14. Bauer, M.; Wunderlich, L.; Weinzierl, F.; Lei, Y.; Duerkop, A.; Alshareef, H.N.; Baeumner, A.J. Electrochemical Multi-Analyte Point-of-Care Perspiration Sensors Using on-Chip Three-Dimensional Graphene Electrodes. *Anal. Bioanal. Chem.* **2021**, *413*, 763–777.
